# Factors in the HIV risk environment associated with bacterial vaginosis among HIV-negative female sex workers who inject drugs in the Mexico-United States border region

**DOI:** 10.1186/s12889-018-5965-9

**Published:** 2018-08-20

**Authors:** Jennifer P. Jain, Claire C. Bristow, Heather A. Pines, Alicia Harvey-Vera, Gudelia Rangel, Hugo Staines, Thomas L. Patterson, Steffanie A. Strathdee

**Affiliations:** 1 Department of Medicine, University of California, San Diego, USA; 2 Facultad de Medicina, Universidad Autónoma de Ciudad Juarez, Ciudad Juárez, MX Mexico; 3 Department of Psychiatry, University of California, San Diego, USA; 4 US-Mexico Border Health Commission, Tijuana, Mexico

**Keywords:** Bacterial vaginosis (BV), Female sex workers who inject drugs (FSW-PWIDs), Mexico, HIV risk environment and gender-based violence

## Abstract

**Background:**

Bacterial vaginosis (BV) is the most common cause of vaginitis among women worldwide and is associated with increased susceptibility to sexually transmitted infections (STIs), including HIV. We aimed to determine the impact of the HIV risk environment on BV among female sex workers who inject drugs (FSW-PWIDs) in Tijuana and Ciudad Juarez, Mexico.

**Methods:**

We performed a cross-sectional analysis utilizing baseline data from a randomized controlled trial evaluating a behavioral HIV prevention intervention. Participants underwent testing for BV using the OSOM BVBlue® Rapid Test (Genzyme Diagnostics, San Diego, CA) and completed a survey eliciting information on the HIV risk environment, sexual risk behaviors, and substance use. We applied logistic regression to identify correlates of BV in the physical, social, economic, and political HIV risk environments stratified by study site (Ciudad Juarez vs. Tijuana).

**Results:**

In total, 584 HIV-negative FSW-PWIDs (300 Ciudad Juarez; 284 Tijuana) were enrolled. The prevalence of BV was 39% (*n* = 228), which was higher in Ciudad Juarez (56.7%) compared to Tijuana (20.4%). In both cities, micro-level components of the physical HIV risk environment were associated with BV. In Ciudad Juarez, BV was associated with past experiences or threats of physical violence in response to proposed condom use (adjusted odds ratio [aOR] = 3.66, 95% confidence interval [CI]: 1.74–7.69, *p* = 0.001) and lifetime residence in Ciudad Juarez (aOR = 1.74, 95% CI: 1.05–2.87, *p* = 0.031). In Tijuana, BV was associated with the number of hours spent on the street daily in the past six months looking for, using, or dealing drugs, engaging in other income generating activities, or sleeping (aOR = 1.05, 95% CI: 1.001–1.097, *p* = 0.045).

**Conclusions:**

Our findings suggest that FSW-PWIDs’ risk of BV may be shaped by the microphysical HIV risk environment. Addressing components of the physical risk environment, including interventions to reduce gender-based violence, may alleviate the burden of BV and subsequent susceptibility to HIV/STIs among FSW-PWIDs in the Mexico/US border region.

**Trial registration:**

National Institute of Health (NIH) Clinical Trials Identifier NCT00840658, and date of NIH trial registration February 7, 2009.

## Background

Bacterial vaginosis (BV) is the most common cause of vaginitis among reproductive-aged women worldwide [1, 2]. BV is characterized by the replacement of lactobacilli with anaerobic bacteria, such as *Gardnerella vaginalis* [3, 4]. BV increases susceptibility to sexually transmitted infections (STIs), including HIV [5–8]. Although the exact role that sexual activity plays remains unclear, BV rarely occurs in its absence suggesting that BV may be a sexually associated condition [4]. While the epidemiology and precise etiology of BV are poorly understood, there are several known correlates of BV including: childbearing age, Hispanic ethnicity, condomless vaginal sex with male and female partners, new and multiple sex partners, intravaginal washing, and infection with HIV or other STIs [3, 9]. Inversely, factors that may protect against BV include condom use during vaginal sex and the use of estrogen or progesterone containing hormonal birth control [1].

BV prevalence varies widely by region and population [6]. In studies of women in the general population in the United States (US), United Kingdom, and Australia, BV prevalence ranged from 9 to 30% [10–12]. However, BV prevalence may be even higher among women who engage in sexual risk behaviors, such as female sex workers (FSWs) [1, 6]. For instance, BV prevalence was estimated to be as high as 45 and 70% among female sex workers (FSWs) in Peru and South Africa, respectively [13, 14].

Many of the aforementioned correlates of BV overlap with HIV/STI risk, thus it is plausible that the HIV risk environment framework as described by Tim Rhodes and colleagues may help illuminate environmental factors associated with BV [15]. The ‘risk environment’ is a conceptual framework that explores how physical, social, political, and economic environments impact overall health and vulnerability to HIV among substance users [15]. Since BV increases the susceptibility to HIV/STIs, it is possible that BV may partially mediate the relationship between the HIV risk environment and HIV acquisition. Therefore, examining BV in the context of the HIV risk environment may further our understanding of the utility of intervening on environmental factors to reduce BV incidence and subsequent HIV/STI risk, especially among highly vulnerable groups such as female sex workers who inject drugs (FSW-PWIDs). We aimed to understand how BV is shaped by the HIV risk environment among FSW-PWIDs in Tijuana and Ciudad Juarez, located along the Mexico/US border.

FSW-PWIDs are considered a uniquely vulnerable population and have disproportionate rates of HIV and STIs [16]. Among FSW-PWIDs in Tijuana and Ciudad Juarez the prevalence of HIV, active syphilis, gonorrhea, and chlamydia is two to three times higher compared to FSWs who do not inject drugs, [16]. These elevated prevalences may be partially due to FSW-PWIDs increased likelihood to concede to the demands for condomless sex, due to experiencing the urgency of drug-related withdrawal or being reliant upon partners or clients for drugs [16, 17]. Further, influences in the economic risk environment such as economic vulnerability, increase FSW-PWIDs susceptibility to demands for condomless sex from clients who pay more for this type of sexual transaction [18]. Taken together, FSW-PWIDs along Mexico’s northern border are at increased risk for HIV/STIs due to a constellation of individual and environmental factors that may also heighten their risk for BV.

The sex work industries in Tijuana and Ciudad Juarez are thriving, attracting clients from Mexico, the US and beyond, making HIV/STI transmission in these regions a global public health issue [19]. The robust nature of this industry is partly due to the regulation of sex work in Mexico [19]. As a result, FSWs are required to obtain a permit and undergo HIV/STI screening every four months, although the majority practice without permits and these procedures do not include screening for BV [19]. In Ciudad Juarez however, the red-light district has been disbanded in recent years forcing FSWs to work underground with limited access to routine HIV/STI testing [19, 20].

The physical risk environment in these cities is largely characterized by their placement along two well-established drug trafficking routes that transport illicit substances into the US frequently [21, 22]. Consequently, the drug markets in these cities are flourishing, and it has been estimated that approximately 18% of FSWs in the region inject drugs [23, 24]. Another defining characteristic of the physical risk environment in these regions is violence [20]. Tijuana and Ciudad Juarez have been subject to severe human rights violations and drug cartel-related violence for decades [20]. This has led to the normalization of violence, and has resulted in a high level of gender-based violence towards FSWs perpetrated by clients, intimate partners, and law enforcement officials [20].

## Methods

### Study setting

We conducted a cross-sectional analysis to characterize the correlates of BV in the physical, social, economic and political HIV risk environments among FSW-PWIDs in Tijuana and Ciudad Juarez, Mexico. In low-income settings such as these, it is crucial to understand where prevention efforts should be targeted in order to effectively prioritize limited resources. In each city study activities took place in private office based settings. In Tijuana, the research site was located near the red light or *“Zona Roja”* district, and in Ciudad Juarez, the research site was located adjacent to downtown or *“El Centro”.*

### Study sample

As previously described, from 2008 to 2010 584 HIV-negative FSW-PWIDs were recruited from known sex work locations and other sites frequented by FSW-PWIDs in Tijuana and Ciudad Juarez for participation in a randomized controlled trial (RCT) designed to evaluate the efficacy of a behavioral HIV prevention intervention [25]. Eligibility criteria included: being biologically female, HIV-negative, at least 18 years of age, reporting exchanging sex for money, drugs, food or shelter in the past month, reporting injection drug use in the past month, reported sharing syringes or injection equipment in the past month, and willing to take antibiotic treatment if they screened positive for gonorrhea, chlamydia or syphilis at baseline [25].

### Ethical considerations

All participants provided written informed consent during which study staff explained the details of confidentiality and the protection of their personal health information. Study procedures were reviewed and approved by Institutional Review Boards at the University of California, San Diego, Centro Nacional para la Prevencion de VIH/SIDA, Universidad Autonoma de Ciudad Juarez, and Hospital General de Tijuana.

### Bacterial vaginosis screening and treatment

Screening and treatment for BV were performed in accordance with the guidelines set forth by Mexico’s Ministry of Health at the time of data collection. Study nurses facilitated the screening process in a private setting using the OSOM BVBlue® Rapid Test (Genzyme Diagnostics, San Diego, CA) [25]. According to prior research assessing the performance of this test, the sensitivity and specificity of the BVBlue® test compared to Gram Stain (Nugent Score) and Amsel criteria were 91.7 and 97.8%, respectively [26]. Women who tested positive for BV were provided free treatment (oral metronidazole), ordered by a medical doctor onsite at the time of diagnosis.

### Measures

Participants completed surveys in a private setting administered by trained interviewers with extensive experience working with FSW-PWIDs in the Mexico/US border region. All staff underwent cultural sensitivity and ethical conduct of research trainings prior to engaging with participants. Furthermore, a psychologist was present onsite at all times to respond to any additional needs or concerns of participants. Surveys collected information on individual-level factors, as well as micro- and macro-level components of the physical, social, economic, and policy HIV risk environment.

#### Individual-level factors

Sociodemographics (age, marital status, number of years of education, ability to speak English), history of sex work and drug use behaviors (age at initiation of sex work, number of years in sex work calculated based on current age and age at initiation of sex work, age at initiation of injection and non-injection drug use), intravaginal washing ever and in the past six months, sexual and reproductive health (history of gynecological exam, non-condom birth control method use in the past six months, including the use of oral contraceptive pills, hormonal injections, patches, vaginal rings, intrauterine devices, and implants), number of male clients in the past month, substance use (frequency of drug and alcohol use before or during sex with clients, binge drinking defined as five or more alcoholic beverages in one sitting in the past month, and risky injection practices (receptive needle sharing, sharing injection paraphernalia, in the past month).

#### Micro-physical risk environment

Living in Tijuana or Ciudad Juarez for one’s whole life, number of hours spent on the street on a typical day in the past six months, including time searching for, using or dealing drugs, engaging in other income generating activities, and sleeping, homelessness in the past month defined as sleeping in a vehicle, abandoned building, shelter or welfare residence, drug treatment center or on the streets, history of arrest, street based sex work based on participants self-identification as a ‘street worker’, history of any rape and physical abuse, exposure to sexual or physical abuse as a child, history of sexual abuse or rape by clients in the past six months, and ever experiencing or being threatened with physical violence from regular clients, non-regular clients or intimate partners when proposing to use a condom for sex of any kind. *Macro-physical risk environment*: history of travel to the US and deportation from the US.

#### Micro-social risk environment

Sexual risk behaviors in the past month (frequency of condom use during vaginal and anal sex with male clients [‘infrequent’ was defined as using condoms never or sometimes vs. always] and self-efficacy towards condom use (4-point Likert scale responses [dichotomized into strongly agree/agree and strongly disagree/disagree] regarding the ability to use condoms: properly, each time one has sex, while under the influence of drugs or alcohol, without any instruction, and whether one can have condoms available each time they have sex) [27].

#### Micro-economic risk environment

Average monthly income of ≥ $3500 pesos [no income, <$1000, $1000–$1499, $1500–$1999, $2000–$2499, $2500–$2999, $3000–$3499 or more than $3500 pesos] the average amount earned for condom-protected and condomless vaginal and anal sex. From these, a dichotomous measure of whether or not women earned more for condomless sex versus condom-protected sex was created. Interactions with law enforcement (i.e. received bribes from police officers in the past six months for sexual favors, money, or syringes in exchange for not being arrested) were also collected.

#### Micro-policy risk environment

History of HIV testing, drug treatment history, and attaining syringes from a needle exchange program in the past month.

### Statistical analyses

Descriptive statistics were used to characterize the study sample with respect to individual-level factors and components of the HIV risk environment by BV status and study site. Bivariate logistic regression was then used to examine whether BV is associated with individual-level factors and physical, social, economic, and political factors that shape the HIV risk environment. Variables with a *p*-value derived from the bivariate logistic regression models of ≤0.20 were considered for inclusion in the final multivariable models. The final models were built using a forward stepwise model building technique. Each independent variable was entered into the model one at a time while controlling for the following confounders that have been identified as correlates of BV in prior research: age in years, average monthly income [≥$3500 Mexican pesos], ever performed intravaginal washing, and the number of male clients in the past month [3, 9, 28]. Variables that did not retain a *p*-value of ≤0.05 were removed from the final multivariable models during the model building process. To examine whether potential correlates of BV differed by geographic region, all analyses were stratified by study site (Ciudad Juarez vs. Tijuana). All statistical analyses were conducted using STATA 14.0 (STATACorp LP, College Station, TX).

## Results

A total of 584 FSW-PWIDs were enrolled, including 300 in Ciudad Juarez and 284 in Tijuana. Participants had a median age of 33 years (IQR = 27, 40) and 37.3% of participants reported being married (Table 1). The median age at first injection was 20 (IQR = 17, 26) and the median age at the initiation of sex work was 19 (IQR = 15, 23). Approximately a quarter of participants 26.9% reported being able to speak English and the median number of years of education completed was 6 (IQR = 5, 9). Overall, BV prevalence was 39%, with a higher percentage of women screening BV positive in Ciudad Juarez compared to Tijuana (52.7% vs. 24.7%; *p* < 0.001).

**Table 1:** Sociodemographic and behavioral characteristics of female sex workers who inject drugs, with and without bacterial vaginosis in Tijuana and Ciudad Juarez, Mexico (*N* = 584)

		Ciudad Juarez (*n* = 300)				Tijuana (*n* = 284)			
Characteristics	Overall (*N* = 584)	BV+ (*n* = 158)	BV- (*n* = 142)	Odd Ratio (95% CI)	Total (*n* = 300)	BV+ (*n* = 70)	BV- (*n* = 214)	Odd Ratio (95% CI)	Total (*n* = 284)
Sociodemographics									
Median age in years (IQR)	33 (27, 40)	32 (26, 38)	33.5 (27, 41)	0.98 (0.95–1.00)	33 (27, 39)	34.5 (29, 42)	33 (27, 41)	1.01 (0.98–1.05)	33.5 (28, 41)
Median number of years of education (IQR)	6 (5, 9)	6 (5, 8)	6 (4, 8)	1.03 (0.94–1.13)	6 (5, 8)	7 (5, 9)	8 (6, 11)	0.92 (0.83–1.00)	8 (6, 10)
Speaks English	157 (26.9)	19 (12.0)	20 (14.1)	0.83 (0.43–1.63)	39 (13%)	27 (38.6)	91 (42.5)	0.85 (0.49–1.47)	118 (41.6%)
Married or in common law marriage	218 (37.3)	56 (35.4)	50 (35.2)	1.01 (0.63–1.62)	106 (35.3%)	33 (47.1)	79 (36.9)	1.52 (0.88–2.63)	112 (39.4%)
Reproductive and sexual health									
Ever had a gynecological exam	108 (18.6)	24 (15.2)	23 (16.2)	0.93 (0.50–1.73)	47 (15.7%)	19 (27.9)	42 (19.6)	1.59 (0.85–2.98)	61 (21.6%)
Non-condom birth control methods^d,a^	229 (39.3)	69 (44.0)	59 (41.6)	1.10 (0.70–1.75)	128 (42.8%)	18 (25.7)	83 (38.8)	0.55 (0.30–0.99)*	101 (35.6%)
Individual risk behaviors lifetime and past six months									
Median age at initiation of sex work (IQR)	19 (15, 23)	18 (16, 23)	18.5 (15, 23)	0.99 (0.95–1.02)	18 (15, 23)	18.5 (15, 24)	19 (15, 23)	1.01 (0.97–1.05)	19 (15, 24)
Median number of years in sex work (IQR)	13 (7, 19)	12 (6, 18)	13 (7, 20)	0.98 (0.96–1.01)	12 (6, 19)	14 (8, 21)	13 (8, 19)	1.01 (0.98–1.04)	13 (7, 19)
Median age at first injection (IQR)	20 (17, 26)	18.5 (16, 27)	20 (17, 28)	0.98 (0.95–1.00)	20 (17, 27)	20 (17, 25)	20 (17, 24)	1.01 (0.97–1.06)	20 (17, 24)
Ever performed intravaginal washing	293 (55.4)	88 (61.5)	90 (68.7)	0.73 (0.44–1.20)	178 (64.9%)	33 (48.5)	82 (43.9)	1.21 (0.69–2.11)	115 (45.1%)
Performed intravaginal washing^d^	229 (43.3)	73 (51.1)	73 (55.7)	0.83 (0.51–1.33)	146 (53.3%)	26 (38.2)	57 (30.5)	1.41 (0.79–2.52)	83 (32.6%)
Individual risk behaviors in the past month									
Median number of male clients (IQR)^e^	30 (10, 80)	69 (36, 104)	68 (30, 108)	1.00 (0.99–1.00)	68 (30, 104)	11 (6, 22)	15 (6, 30)	0.99 (0.98–1.01)	15 (6, 20)
Any receptive needle sharing^e^	561 (96.2)	152 (96.2)	138 (97.2)	0.73 (0.20–2.66)	290 (96.7%)	68 (97.1)	203 (95.3)	1.67 (0.36–7.83)	271 (95.8%)
Shared injection paraphernalia ≥ half of the time^e,b^	362 (62.2)	86 (54.4)	79 (55.6)	0.95 (0.60–1.50)	165 (55%)	52 (75.4)	145 (68.1)	1.43 (0.77–2.66)	197 (69.9%)
Drug use before or during sex^e^	531 (91.1)	144 (91.1)	135 (95.1)	0.53 (0.21–1.36)	279 (93%)	60 (85.7)	192 (90.1)	0.66 (0.29–1.47)	252 (89.1%)
Alcohol use before or during sex^e^	296 (50.8)	99 (62.7)	93 (65.5)	0.88 (0.55–1.42)	192 (64%)	24 (34.3)	80 (37.6)	0.87 (0.49–1.53)	104 (36.8%)
Binge drinking^e,c^	271 (46.5)	93 (58.9)	94 (66.2)	0.73 (0.46–1.17)	187 (62.3%)	19 (27.1)	65 (30.5)	0.85 (0.46–1.55)	84 (29.7%)

Some percentages are based on denominators smaller than the N listed in the column heading, this is due to missing data

*P*-value, * < 0.05

^a^Non-condom birth control method use was defined by using any of the following types of birth control: pill, hormone injection, patch, vaginal ring, intrauterine device, implant

^b^Injection paraphernalia includes bottle caps/cookers and cottons

^c^Binge drinking defined as having five or more alcoholic drinks in one sitting

^d^Past 6 months

^e^Past month

FSW-PWIDs differed by site with respect to certain individual level risk factors for BV. The median number of male clients in the past month reported by women in Ciudad Juarez was higher than the median number reported by women in Tijuana (median = 68; IQR = 30, 104 vs. median = 15; IQR = 6, 30, < 0.001) respectively. A larger percentage of women in Ciudad Juarez reported intravaginal washing in the past six months compared to women in Tijuana (53.3% vs. 32.6%, *p* < 0.001).

Women in Ciudad Juarez and Tijuana differed with respect to the microphysical HIV risk environment. Compared to Tijuana, a greater percentage of participants from Ciudad Juarez reported ever being physically abused (63% vs. 33%; *p <* 0.001), ever-experiencing client-perpetrated violence (39.3% vs. 20.7%, *p <* 0.001), and being raped by a client in the past 6 months (27% vs. 17%, *p* < 0.01). Finally, a greater percentage of women in Ciudad Juarez identified the street as their primary work environment (91.3% vs. 82.8%, *p <* 0.01).

In our unadjusted analysis of FSW-PWIDs in Tijuana, BV was negatively associated with non-condom birth control methods (OR = 0.55, 95% CI = 0.30–0.99) and positively associated with the number of hours spent on the street (OR = 1.05, 95% CI = 1.01–1.09) (Tables 1 and 2). In Ciudad Juarez, BV was positively associated with lifetime residence in Ciudad Juarez (OR = 1.69, 95% CI = 1.07–2.67) and experiencing or being threatened with physical violence in response to the proposition of condom use (OR = 2.68, 95% CI = 1.40–5.12).

**Table 2:** HIV risk environment factors associated with bacterial vaginosis among female sex workers who inject drugs in Tijuana and Ciudad Juarez, Mexico (*N* = 584)

		Ciudad Juarez (*n* = 300)				Tijuana (*n* = 284)			
Characteristics	Overall (*N* = 584)	BV+ (*n* = 158)	BV- (*n* = 142)	Odds Ratio (95% CI)	Total (*n* = 300)	BV+ (*n* = 70)	BV- (*n* = 214)	Odds Ratio (95% CI)	Total (*n* = 284)
Physical Risk Environment									
*Micro-physical*									
Lifetime residence in home city	251 (43.0)	94 (59.5)	66 (46.5)	1.69 (1.07–2.67)*	160 (53.3%)	25 (35.7)	66 (30.8)	1.25 (0.71–2.20)	91 (32%)
Street based sex work^a^	509 (87.2)	146 (92.4)	128 (90.1)	1.33 (0.59–2.98)	274 (91.3%)	56 (80.0)	179 (83.6)	0.78 (0.39–1.56)	235 (82.8%)
Median # hours spent on the street (IQR)^i,b^	10 (7, 15)	9 (7, 12)	10 (7, 12)	0.99 (0.94–1.04)	9 (7, 12)	15 (10, 20)	12 (7, 18)	1.05 (1.01–1.09)*	12 (8, 18)
Mostly homeless^j,c^	35 (6.0)	4 (2.5)	5 (3.5)	0.71 (0.19–2.70)	9 (3%)	8 (11.4)	18 (8.4)	1.41 (0.58–3.39)	26 (9.2%)
Ever raped	293 (50.7)	99 (62.7)	85 (59.9)	1.13 (0.71–1.79)	184 (61.3%)	24 (34.8)	85 (40.7)	0.78 (0.44–1.37)	109 (39.2%)
Sexually abused/raped as a child	194 (33.6)	66 (41.8)	60 (42.3)	0.98 (0.62–1.55)	126 (42%)	15 (22.1)	53 (25.4)	0.83 (0.43–1.60)	68 (24.6%)
Sexually abused/raped by client^i^	129 (22.6)	46 (29.5)	35 (24.8)	1.27 (0.76–2.12)	81 (27.3%)	14 (20.6)	34 (16.4)	1.32 (0.66–2.64)	48 (17.5%)
Ever physically abused	280 (48.5)	100 (63.3)	89 (62.7)	1.03 (0.64–1.64)	189 (63%)	23 (33.8)	68 (32.5)	1.06 (0.59–1.89)	91 (32.9%)
Physically abused as a child	140 (24.3)	54 (34.2)	41 (28.9)	1.28 (0.78–2.09)	95 (31.7%)	9 (13.4)	36 (17.2)	0.75 (0.34–1.64)	45 (16.3%)
Ever been arrested	434 (74.6)	125 (79.1)	119 (83.8)	0.73 (0.41–1.32)	244 (81.3%)	51 (72.9)	139 (65.6)	1.41 (0.78–2.56)	190 (67.4%)
Ever experienced physical violence or a threat of physical violence when proposing to use a condom^d^	90 (15.4)	38 (24.1)	15 (10.6)	2.68 (1.40–5.12)**	53 (17.7%)	9 (12.9)	28 (13.2)	0.97 (0.44–2.18)	37 (13.1%)
*Macro-physical*									
Ever traveled to the United States	300 (51.4)	70 (44.3)	73 (51.4)	0.75 (0.48–1.18)	143 (47.7%)	36 (51.4)	121 (56.5)	0.81 (0.47–1.40)	157 (55.3%)
Ever deported from the United States	51 (8.8)	7 (4.4)	3 (2.1)	2.13 (0.54–8.41)	10 (3.3%)	8 (11.4)	33 (15.9)	0.68 (0.30–1.56)	41 (14.8%)
Social Risk Environment									
*Micro-social*									
Infrequent condom use with clients for vaginal sex^j,e^	250 (42.9)	100 (63.3)	87 (61.7)	1.07 (0.67–1.71)	187 (62.5%)	17 (24.3)	46 (21.6)	1.16 (0.62–2.20)	63 (22.3%)
Infrequent condom use with clients for anal sex^j,e^	102 (22.4)	32 (29.9)	33 (30.8)	0.96 (0.53–1.71)	65 (30.4%)	4 (7.6)	33 (17.5)	0.39 (0.13–1.14)	37 (15.3%)
Median number of condomless vaginal sex acts with clients (IQR)^j^	29 (9, 57)	28 (11, 60)	40 (12, 70)	1.00 (0.99–1.00)	32.5 (12, 64)	19.5 (7.5, 49.5)	27 (5.5, 50)	1.00 (0.99–1.00)	25 (6, 50)
Reports being able to use a condom properly	491 (85.2)	140 (89.7)	124 (88.6)	1.13 (0.54–2.35)	264 (89.2)	61 (88.4)	166 (78.7)	2.07 (0.92–4.63)	227 (81.1)
Reports being able to use a condom each time they have sex	484 (83.6)	139 (88.5)	121 (86.4)	1.21 (0.61–2.42)	260 (87.5%)	58 (84.06)	166 (77.9)	1.49 (0.73–3.07)	224 (79.4%)
Reports being able to have condoms available each time they have sex	391 (67.3)	87 (55.1)	85 (60.3)	0.81 (0.51–1.28)	172 (57.5%)	54 (78.3)	165 (77.5)	1.05 (0.54–2.02)	219 (77.7%)
Reports being able to use a condom under the influence of drugs or alcohol	376 (64.8)	93 (59.2)	88 (62.4)	0.88 (0.55–1.40)	181 (60.7%)	50 (71.4)	145 (68.4)	1.16 (0.64–2.10)	195 (69.2%)
Reports being able to use a condom without any instruction	473 (81.6)	134 (84.8)	124 (87.9)	0.77 (0.39–1.49)	258 (86.3%)	57 (82.6)	158 (74.5)	1.62 (0.81–3.25)	215 (76.5%)
Economic Risk Environment									
*Micro-economic*									
Monthly average income of ≥ $3500 pesos or $350 USD^f^	276 (47.6)	96 (60.8)	84 (59.2)	1.07 (0.67–1.70)	180 (60%)	19 (27.5)	77 (36.5)	0.66 (0.36–1.20)	96 (34.3%)
Median average amount earned per condom-protected sex transaction^g^	15 (10, 20)	10 (10,15)	10 (10, 15)	1.00 (0.96–1.05)	10 (10, 15)	20 (20, 30)	20 (20, 30)	1.01 (1.00–1.01)	20 (20, 30)
Median average amount earned per condomless sex transaction^g^	20 (15, 30)	15 (10, 20)	15 (10, 25)	0.99 (0.97–1.02)	15 (10, 20)	20 (20, 40)	25 (20, 35)	1.00 (0.99–1.01)	25 (20, 40)
Reported earning more for condomless sex^i^	269 (47.4)	97 (62.2)	89 (63.6)	0.94 (0.59–1.51)	186 (62.8%)	19 (28.4)	64 (31.4)	0.87 (0.47–1.59)	83 (30.6%)
Police solicited bribes instead of arrest^i,h^	360 (61.9)	121 (76.6)	95 (66.9)	1.62 (0.97–2.69)	216 (72%)	38 (54.3)	106 (50.0)	1.19 (0.69–2.04)	144 (51.1%)
Policy Risk Environment									
*Micro-policy*									
Self-reported ever being tested for HIV	292 (50.1)	77 (48.7)	71 (50.0)	0.95 (0.60–1.50)	148 (49.3%)	40 (57.1)	104 (48.8)	1.40 (0.81–2.41)	144 (50.9%)
Attended a needle exchange program^j^	60 (10.3)	16 (10.1)	18 (12.8)	0.77 (0.38–1.57)	34 (11.4%)	9 (12.9)	17 (8.0)	1.69 (0.72–3.99)	26 (9.2%)
Ever enrolled in drug treatment	297 (50.9)	100 (63.3)	78 (54.9)	1.41 (0.89–2.25)	178 (59.3%)	31 (44.3)	88 (41.3)	1.13 (0.65–1.95)	119 (42.1%)

Some percentages are based on denominators smaller than the N listed in the column heading, this is due to missing data

*P*-value, * < 0.05, ** < 0.01

^a^Street based sex work is defined by women who used “street worker” to describe their work situation

^b^IQR = Inter-quartile range. Median number of hours spent on the street on a typical day in the past six months including time looking for or dealing drugs, performing other activities to obtain money, using drugs and sleeping on the street

^c^Mostly homeless is defined as sleeping in: a vehicle, abandoned building, shelter or welfare residence, drug treatment center or on the streets

^d^Ever experienced physical violence or received a threat of physical violence from regular clients, non-regular clients or intimate partners in response to the proposition of condom use for sex of any kind

^e^Used condoms never or ‘sometimes’ in the past month

^f^USD, calculated with the exchange rate from 2008 when the data was collected (1 USD = 10 pesos)

^g^USD

^h^Sexual favors, sexual abuse, syringes confiscated and or money taken in exchange for arrest

^i^Past 6 months

^j^Past month

After adjusting for potential confounders, the positive associations between BV and components of the microphysical HIV risk environment remained in both Ciudad Juarez and Tijuana (Table 3). In Ciudad Juarez, BV was associated with experiencing or being threatened with physical violence in response to the proposition of condom use (aOR = 3.66, 95% CI = 1.74–7.69, *p* = 0.001) and lifetime residence in Ciudad Juarez (aOR = 1.74, 95% CI = 1.05–2.87, *p* = 0.031). Finally, for women in Tijuana BV was associated with the number of hours spent on the street, such that for every one-hour increase spent on the street the odds of BV increased by 5% (aOR = 1.05, 95% CI = 1.001–1.097, *p* = 0.045).

**Table 3:** Factors in the hiv risk environment associated with bacterial vaginosis among female sex workers who inject drugs in Ciudad Juarez and Tijuana, Mexico (*N* = 584)

Exposure	Ciudad Juarez (*n* = 300) aOR (95% CI)	*P*-value	Tijuana (*n* = 284) aOR (95% CI)	*P*-value
*Micro-Physical Risk Environment*				
Ever experienced violence when proposing to use a condom^b^	3.66 (1.74–7.69)**	0.001**		
Lifetime residence in home city	1.74 (1.05–2.87)*	0.031*		
Median number of hours spent on the street^a,c^			1.05 (1.001–1.097)	0.045*

Controlled for: age in years, monthly average income of ≥ $350 USD ($3500 pesos), ever performed intravaginal washing and reported number of male clients in the past month

*P*-values derived from Logistic Regression, * < 0.05, ** < 0.01

^a^Past 6 months

^b^Violence from regular clients, non-regular clients and partners

^c^Median number of hours spent on the street includes time looking for drugs, using drugs and sleeping on the street

## Discussion

We examined the impact of different levels and aspects of the HIV risk environment on BV among FSW-PWIDs in the Mexico/US border region. This work suggests that microphysical environmental influences may have a negative impact on sexual and reproductive health outcomes in this population. This may be primarily due to constraining FSW-PWIDs ability to engage in protective behaviors against BV, which could in turn mediate subsequent risk for HIV/STIs among FWS-PWIDs in this region.

The elevated prevalence of BV among participants in Ciudad Juarez compared to Tijuana implies that underlying contextual factors unique to Ciudad Juarez may be driving BV prevalence in that setting. For instance, the decentralization of the red-light district in Ciudad Juarez has resulted in the deregulation of the sex work industry, which has displaced FSWs to truck stops, residential zones, and the streets [20, 22]. Prior research among FSWs in Ciudad Juarez demonstrated that sex work venue instability and sex work performed outdoors is associated with decreased condom use [22]. We found that a greater percentage of FSW-PWIDs in Ciudad Juarez compared to Tijuana identified the street as their primary work location. Further, our findings demonstrated a higher percentage of women in Ciudad Juarez that reported intravaginal washing in the past six months and a higher median number of male clients in the past month. Notably, the median number of male clients in the past month reported by women in Ciudad Juarez was over five times greater than the median for women Tijuana. These factors may partially explain the positive association between lifetime residence in Ciudad Juarez and BV, indicating that prolonged exposure to contextual factors in this city influence FSW-PWIDs risk behaviors that potentiate poor sexual and reproductive health outcomes.

The association between physical violence or threats of physical violence in response to the proposition of condom use and BV among FSW-PWIDs in Ciudad Juarez adds to the existing body of literature documenting the impact of gender-based violence on sexual and reproductive health among FSW-PWIDs in the Mexico-US border region. A longitudinal study assessing factors that affect HIV/STI transmission among FSWs in Tijuana and Ciudad Juarez found that client-perpetrated violence was correlated with street-based sex work and inconsistent condom use with non-regular clients [20]. Further, client-perpetrated violence was associated with a perceived decrease in sexual relationship power, which contributes to a compromised ability to negotiate condom use [20, 29]. In our sample, client-perpetrated violence, being raped by a client in the past six months and ever being physically abused were all more common among FSW-PWIDs in Ciudad Juarez. These findings suggest that interventions should target the underlying gender inequities that fuel power inequalities intrinsic to the risk negotiation process. In doing so, prevention efforts may mitigate the impact of the microphysical environment on BV and subsequent HIV/STIs risk among FSW-PWIDs in Ciudad Juarez.

In Tijuana, the association between the average number of hours spent on the street looking for, using, or dealing drugs, performing other activities to obtain money, or sleeping and BV has several important public health implications. This association may capture the impact of street-based sex work, economic marginalization, and increased dependence on illicit substances on the reproductive health of FSW-PWIDs. First, FSW-PWIDs who spend more time on the street engaging in any of the aforementioned activities may have fewer resources (e.g., stable housing) and engage in street-based sex work more compared to other FSW-PWIDs. According to prior research that compared HIV risk behaviors between street-based FSWs and FSWs who work in bars in Tijuana, those who worked on the street were at increased risk for HIV/STIs due to the combined impact of infrequent condom use, greater access to illicit substances, more illicit drug use, and a larger client load [30]. Notably, infrequent condom use was associated with reduced access to condoms and HIV prevention services, increased economic incentives for condomless sex and decreased efficacy to use condoms [30]. Taken together, this finding is consistent with the implications of the aforementioned associations between BV and women in Ciudad Juarez which highlight the need to address structural and microenvironmental factors in interventions for FSW-PWIDs in the Mexico/US border region.

Findings from this study should be interpreted in the context of several limitations. First, data were collected from a subgroup of FSW-PWIDs along the Mexico/US border, that were recruited using non-random sampling techniques which may limit the generalizability of these results to FSW-PWIDs in other settings. Data were collected between 2008 and 2010 and therefore may not be representative of current trends. For instance, changes in the sex work industry in Ciudad Juarez due to gentrification over the last decade may have altered the HIV risk environment. Thus, more research is needed to ensure that future interventions are responding to changing risk environments. This study relies on self-reported information of sensitive behaviors (e.g. sex work), which may have led to underreporting of behaviors considered socially undesirable. This analysis used cross-sectional data, we cannot disentangle the temporality of measured association, and therefore cannot infer causal relationships.

Our study provides important information regarding the factors in the HIV risk environment that are associated with BV. Our findings highlight the need for comprehensive interventions that address the sociostructural barriers, which prevent FSW-PWIDs from practicing sex work in safe environments and attaining sexual and reproductive health*.* Further, it illuminates the need to focus intervention efforts on micro level factors in the physical risk environment including; policy and cultural standards as they relate to violence targeted toward FSW-PWIDs. In conclusion, this study offers further evidence that health is a product of social structures and environments, and recommends amendments to the political, legal, and cultural contexts that influence the health of FSW-PWIDs in Ciudad Juarez and Tijuana, Mexico.

## Abbreviations

BV: Bacterial vaginosis
FSW-PWIDs: Female sex workers who inject drugs
FSWs: Female sex workers
HIV: Human immunodeficiency virus
STIs: Sexually transmitted infections
US: United States

## References

[1] Guedou FA, Van Damme L, Deese J, Crucitti T, Becker M, Mirembe F (2013). Behavioural and medical predictors of bacterial vaginosis recurrence among female sex workers: longitudinal analysis from a randomized controlled trial. BMC Infect Dis.

[2] Bradshaw CS, Walker J, Fairley CK, Chen MY, Tabrizi SN, Donovan B (2013). Prevalent and incident bacterial vaginosis are associated with sexual and contraceptive behaviours in young Australian women. PloS One.

[3] Bautista CT, Wurapa E, Sateren WB, Morris S, Hollingsworth B, Sanchez JL (2016). Bacterial vaginosis: a synthesis of the literature on etiology, prevalence, risk factors, and relationship with chlamydia and gonorrhea infections. Mil Med Res.

[4] Nasioudis D, Linhares IM, Ledger WJ, Witkin SS (2017). Bacterial vaginosis: a critical analysis of current knowledge. BJOG.

[5] Marrazzo JM (2011). Interpreting the epidemiology and natural history of bacterial vaginosis: are we still confused?. Anaerobe.

[6] Cohen CR, Lingappa JR, Baeten JM, Ngayo MO, Spiegel CA, Hong T (2012). Bacterial vaginosis associated with increased risk of female-to-male HIV-1 transmission: a prospective cohort analysis among African couples. PLoS Med.

[7] Gallo MF, Macaluso M, Warner L, Fleenor ME, Hook EW, Brill I (2012). Bacterial vaginosis, gonorrhea, and chlamydial infection among women attending a sexually transmitted disease clinic: a longitudinal analysis of possible causal links. Ann Epidemiol.

[8] Gillet E, Meys JF, Verstraelen H, Bosire C, De Sutter P, Temmerman M (2011). Bacterial vaginosis is associated with uterine cervical human papillomavirus infection: a meta-analysis. BMC Infect Dis.

[9] Kenyon C, Colebunders R, Crucitti T (2013). The global epidemiology of bacterial vaginosis: a systematic review. Am J Obstet Gynecol.

[10] Allsworth JE, Peipert JF (2007). Prevalence of bacterial vaginosis: 2001-2004 National Health and nutrition examination survey data. Obstet Gynecol.

[11] Lamont RF, Morgan DJ, Wilden SD, Taylor-Robinson D (2000). Prevalence of bacterial vaginosis in women attending one of three general practices for routine cervical cytology. Int J STD AIDS.

[12] Fethers KA, Fairley CK, Morton A, Hocking JS, Hopkins C, Kennedy LJ (2009). Early sexual experiences and risk factors for bacterial vaginosis. J Infect Dis.

[13] Johnson LF, Coetzee DJ, Dorrington RE (2005). Sentinel surveillance of sexually transmitted infections in South Africa: a review. Sex Transm Infect.

[14] Perla ME, Ghee AE, Sanchez S, McClelland RS, Fitzpatrick AL, Suarez-Ognio L (2012). Genital tract infections, bacterial vaginosis, HIV, and reproductive health issues among Lima-based clandestine female sex workers. Infect Dis Obstet Gynecol.

[15] Rhodes T (2002). The 'risk environment': a framework for understanding and reducing drug-related harm. Int J Drug Policy.

[16] Strathdee SA, Philbin MM, Semple SJ, Pu M, Orozovich P, Martinez G (2008). Correlates of injection drug use among female sex workers in two Mexico-U.S. border cities. Drug Alcohol Depend.

[17] Lau JT, Tsui HY, Zhang Y, Cheng F, Zhang L, Zhang J (2008). Comparing HIV-related syringe-sharing behaviors among female IDU engaging versus not engaging in commercial sex. Drug Alcohol Depend.

[18] Robertson AM, Syvertsen JL, Amaro H, Martinez G, Rangel MG, Patterson TL (2014). Can’t Buy My Love: A Typology of Female Sex Workers’ Commercial Relationships in the Mexico–U.S. Border Region. J Sex Res.

[19] Strathdee SA, Lozada R, Martinez G, Vera A, Rusch M, Nguyen L (2011). Social and structural factors associated with HIV infection among female sex workers who inject drugs in the Mexico-US border region. PloS One.

[20] Conners EE, Silverman JG, Ulibarri M, Magis-Rodriguez C, Strathdee SA, Staines-Orozco H (2016). Structural Determinants of Client Perpetrated Violence Among Female Sex Workers in Two Mexico-U.S. Border Cities. AIDS Behav.

[21] Burgos JL, Patterson TL, Graff-Zivin JS, Kahn JG, Rangel MG, Lozada MR (2016). Cost-Effectiveness of Combined Sexual and Injection Risk Reduction Interventions among Female Sex Workers Who Inject Drugs in Two Very Distinct Mexican Border Cities. PloS One.

[22] Gaines TL, Rudolph AE, Brouwer KC, Strathdee SA, Lozada R, Martinez G (2013). The longitudinal association of venue stability with consistent condom use among female sex workers in two Mexico–USA border cities. Int J STD AIDS.

[23] Patterson TL, Semple SJ, Fraga M, Bucardo J, De la Torre A, Salazar-Reyna J (2006). A sexual risk reduction intervention for female sex workers in Mexico. J HIV/AIDS Soc Serv.

[24] Patterson TL, Semple SJ, Staines H, Lozada R, Orozovich P, Bucardo J (2008). Prevalence and correlates of HIV infection among female sex workers in 2 Mexico-US border cities. J Infect Dis.

[25] Vera A, Abramovitz D, Lozada R, Martinez G, Rangel MG, Staines H (2012). Mujer Mas Segura (Safer Women): a combination prevention intervention to reduce sexual and injection risks among female sex workers who inject drugs. BMC Public Health.

[26] Myziuk L, Romanowski B, Johnson SC (2003). BVBlue Test for Diagnosis of Bacterial Vaginosis. J Clin Microbiol.

[27] Patterson TL, Mausbach B, Lozada R, Staines-Orozco H, Semple SJ, Fraga-Vallejo M (2008). Efficacy of a Brief Behavioral Intervention to Promote Condom Use Among Female Sex Workers in Tijuana and Ciudad Juarez, Mexico. Am J Public Health.

[28] Bilardi JE, Walker SM, Temple-Smith MJ, McNair RP, Mooney-Somers J, Vodstrcil LA (2017). Women view key sexual behaviours as the trigger for the onset and recurrence of bacterial vaginosis. PloS One.

[29] Ulibarri MD, Strathdee SA, Lozada R, Magis-Rodriguez C, Amaro H, O’Campo P (2010). Intimate partner violence among female sex workers in two Mexico-U.S. border cities: partner characteristics and hiv risk-behaviors as correlates of abuse. Psychological Trauma.

[30] Larios SE, Lozada R, Strathdee SA, Semple SJ, Roesch S, Staines H (2009). An exploration of contextual factors that influence HIV risk in female sex workers in Mexico: The Social Ecological Model applied to HIV risk behaviors. AIDS Care.

